# A Look Inside—Histopathological Examinations of Different Tail Tip Lesions in Dairy Cows

**DOI:** 10.3390/ani14142094

**Published:** 2024-07-17

**Authors:** Lea M. Lorenz, Marielle E. Volkwein, Christine Schmidt, Mirjam Lechner, Prisca V. Kremer-Rücker

**Affiliations:** 1 Animal Health and Welfare in Livestock Breeding, Faculty of Agriculture, Food and Nutrition, Hochschule Weihenstephan-Triesdorf, University of Applied Sciences, 91746 Weidenbach-Triesdorf, Germany; 2 Aulendorf State Veterinary Diagnostic Centre, 88326 Aulendorf, Germany; 3 Unabhängige Erzeugergemeinschaft Hohenlohe Franken, 97996 Niederstetten-Adolzhausen, Germany

**Keywords:** tail inflammation and necrosis, tail lesions, dairy cows, histopathology

## Abstract

**Simple Summary:**

Lesions of the tail have been observed in fattening cattle for several decades. Ultimately, the tail tips become inflamed and die off. Thus, these lesions impair animal welfare and lead to economic losses. However, little attention has been paid to this issue in dairy cows. In a previous study, we showed that dairy cows are also affected by a wide variety of different lesions of the tails and that these lesions are quite common. However, the cause of these lesions is unclear to date. To reduce the occurrence of these lesions in the future, it is essential to identify their cause. Therefore, we examined affected tails both macroscopically and microscopically in order to gain insight into the underlying pathological processes. Our results point towards reduced blood flow as the cause of the necrosis of the tail tips. This hypothesis should therefore be investigated and tested in future studies.

**Abstract:**

Feedlot cattle are frequently affected by inflammation and necrosis of the tail tips, resulting in impeded animal welfare and economic losses. In a recent study, it was demonstrated that dairy cows are also affected by different lesions of the tail tip, including alopecia, annular constrictions, crusting, scaling and swelling. Despite the frequent occurrence of these lesions, the underlying etiology and pathomechanisms are unclear to date. To gain insight into this malady, we histopathologically examined 16 tail tips of slaughtered dairy cows, representing the entire range of different lesions. While macroscopically alopecic areas were characterized by the formation of granulation tissue in the dermis with an inconspicuous epidermis, we found not only dermal granulation tissue but also purulent-necrotizing inflammation with the breakdown of the basement membrane in encrusted lesions. Interestingly, in some cases, we found areas of coagulation necrosis of the epidermal and dermal layers in the crusts. Tails with macroscopical scaling were affected by ortho- and/or parakeratotic hyperkeratosis, and, in tails with macroscopical swelling, we observed a low-protein edema of the central longitudinal connective tissue of the tail. We conclude that the observed lesions might be caused by ischemia of the skin in the distal parts of the tail.

## 1. Introduction

Tail inflammation and necrosis is a well-known and highly prevalent problem in housed fattening cattle, resulting in impeded animal welfare and economic losses [1,2,3]. The gross lesions range from hairless areas of the tail tip, laceration and infection with crust formation to phlegmonous areas with suppuration affecting not only the tip but also larger parts of the tail [1]. While slatted floors, the increasing weight of fattening bulls and decreased space allotment were previously identified as risk factors [4], the exact etiology and pathophysiological mechanisms underlying this malady remain unknown [1]. The histopathological picture is characterized by hyperkeratosis, acanthosis and dermal fibrosis in tails with alopecia and crustiness. In milder cases, only vessel wall and perivascular edema, as well as perivascular hemorrhage, are described [1].

In contrast to fattening cattle, there are few data on this issue in dairy cows, but there is growing interest. A recent study on dairy cows in New Zealand investigated the tails concerning deviation, trauma/swelling and shortening and found a median prevalence of tails with damage due to deviations and/or trauma of 11.5% [5]. A Danish paper described so-called band-shaped lesions in Holstein cows. The histopathological examination of these lesions revealed granulation tissue with the loss of hair follicles and sebaceous glands of the skin, indicating secondary healing. Furthermore, the affected lesions showed epidermal hyperplasia and hyperkeratosis [6]. In a previous study, we focused on the distal, invertebrate parts of the tails of dairy cows and developed an overview outlining the different tail lesions in more detail [7]. Among the observed lesions were alopecia, hemorrhages and necrosis of the very tip of the tail, as well as annular constrictions and even the rupture or partial loss of the tail. Further findings were scaling, swelling or thinning of the tails and wart-like masses [7]. The overall frequency regarding all types of lesions ranged between 84.0% and 94.1% in the examined Holstein herds and between 97.0% and 99.0% in Simmental herds [7]. To our knowledge, there are no detailed histopathological studies investigating the wide variety of tail lesions in dairy cows.

Therefore, the aim of the present study was to perform a histopathological examination of all of the macroscopically observable tail lesions in dairy cows as a means to better assess their cause and pathogenesis.

## 2. Materials and Methods

For this purpose, we collected the tails of slaughtered dairy cows at a South German abattoir in May 2022. Sixteen tail tips with 59 observed gross lesions in total were selected after macroscopical examination and transported on ice to the State Veterinary Diagnostic Centre—Aulendorf for examination. The purpose of the preselection was to cover the entire range of macroscopical tail alterations described previously [7]. The selected tails originated from dairy cows representing the breeds Brown Swiss (eight animals), Simmental (three animals), Holstein (two animals), a crossbreed of beef and dairy cattle (two animals) and one unknown breed. After transportation, the tail tips were shorn and washed. Subsequently, macroscopical lesions were photodocumented and assessed. The criteria of the evaluation were annular constrictions; lesions at the very tip of the tail, such as hairless or encrusted areas; scaling; swelling; the narrowing of the tail; and wart-like masses. The examination was focused on the distal, invertebrate part of the tail. Therefore, changes in vertebrae were not investigated in this study. For the histopathological examination, the observed lesions were fixed in 10% buffered formalin within twenty-four hours of sampling for at least four days. Subsequently, they were embedded in paraffin, processed routinely for histologic examination and stained with hematoxylin and eosin (H.E.). Selected sections were also stained via the periodic acid–Schiff reaction. The slides were examined using light microscopy.

## 3. Results

The macroscopic examination of the tail tips revealed that some samples were affected by several lesions. In total, we observed 59 different gross lesions in 16 tail tips (overview given in Table 1).

### 3.1. Annular Constrictions

**Figure 1:**
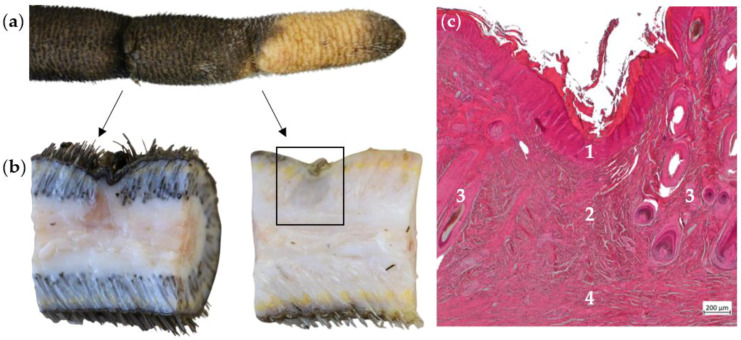
Annular constrictions with alopecic but otherwise intact skin. (**a**) Distal part of a tail with two annular constrictions. The more proximal constriction encompasses about 95% of the tail circumference and the more distal constriction about 50%. The skin in these areas is alopecic but otherwise intact. (**b**) Sagittal sections through the constrictions. In both cases, the epidermis is inconspicuous. No hair follicles can be seen in the dermis underneath the constriction. (**c**) Histopathological findings of the area marked in (**b**), H.E. The epidermis (1) is without pathological findings. The dermis in the affected area is characterized by granulation tissue (2), which contains no hair follicles or sebaceous glands, in contrast to the adjacent area (3). The granulation tissue extends down to the central longitudinal connective tissue of the tail (4).

We found annular constrictions in ten tails, of which seven tails had multiple constrictions. Macroscopically, the constrictions were up to five mm deep at the deepest point, encompassing between 20% and 95% of the tail circumference. In the majority of cases, the skin texture at the deepest part of the constriction was alopecic but otherwise intact (14 constrictions, Figure 1a,b).

**Figure 2:**
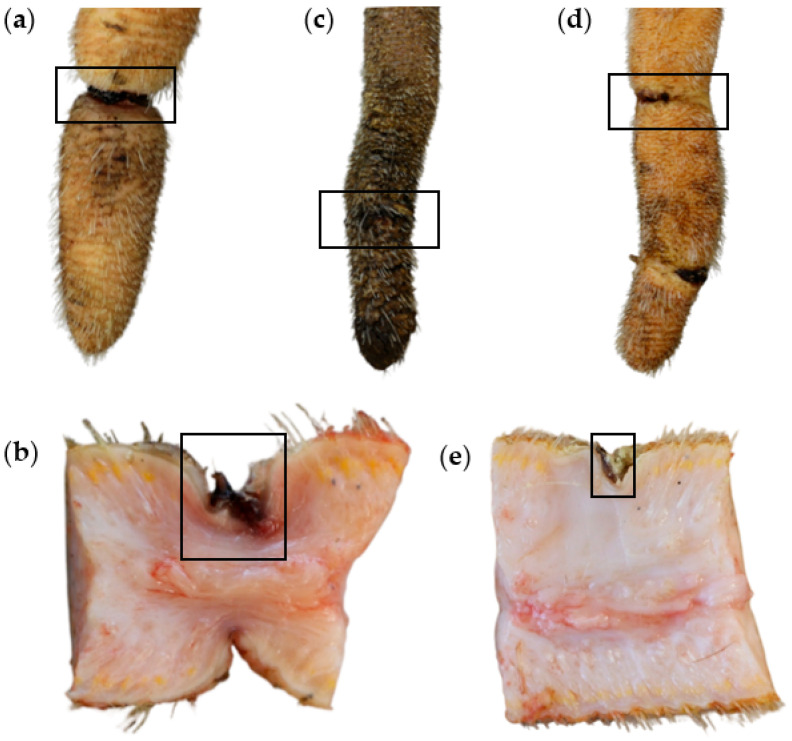
Annular constrictions with crust formation. (**a**) Distal part of a tail with an annular constriction encompassing the entire circumference of the tail. The center of the constriction is bloody encrusted. (**b**) Sagittal section through the annular constriction shown in (**a**). In the marginal area, the skin is alopecic and no hair follicles or sebaceous glands can be found in the area under the constriction. In the central area of the constriction, there are bloody crusts. (**c**) Distal part of a tail with annular constriction encompassing about 70% of the tail. The constriction is bloody encrusted. The tail tip is also affected by severe scaling. (**d**) Distal part of a tail with two annular constrictions with crust formation. The more proximal constriction encompasses about 75% of the tail circumference. (**e**) Sagittal section of the proximal constriction shown in (**d**). The constricted area is devoid of hair follicles or sebaceous glands. In the center of the constriction, the epidermis is covered by crusts.

In all cases of annular constriction, the histopathological examination revealed granulation tissue up to fibrosis underneath the epidermis, extending to the central longitudinal connective tissue of the tail (Figure 1c). Hair follicles and their appendages were not detectable in the affected areas (Figure 1b,c). The epidermis was affected by orthokeratotic hyperkeratosis in four and predominantly parakeratotic hyperkeratosis in two constrictions with macroscopically intact skin. Two constrictions were bloody encrusted, and four constrictions showed both hairless skin in the marginal areas and bloody crusts centrally in the area of the constriction (Figure 2).

**Figure 3:**
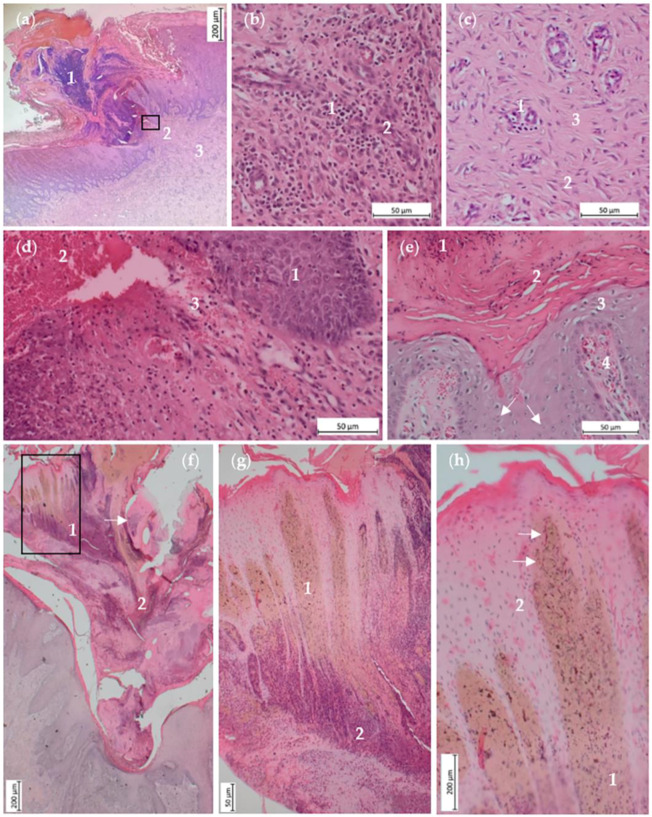
Histological sections of annular constrictions with crust formation, H.E. (**a**) Histological section of the constricted area marked in 2 (**a**) and 2 (**b**). Purulent-necrotizing inflammation of the epidermis with crusting (1), breakdown of the epidermal basement membrane (2) and underlying granulation tissue (3). (**b**) Magnification of the granulation tissue depicted in (**a**) directly underlying the lesion. Accumulation of mononuclear cells (1) and sprouting new blood vessels (2). (**c**) Magnification of the granulation tissue depicted in (**a**) in the deeper layers underneath the lesion, containing blood vessels (1), fibrocytes (2) and collagen (3). (**d**) Magnification of the area marked in (**a**) with spongiosis (1), hemorrhage (2) and the breakdown of the basement membrane (3). (**e**) Histological section of the constricted area marked in 2 (**c**), showing the accumulation of neutrophil granulocytes (1), parakeratotic hyperkeratosis (2) and intracellular edema of keratinocytes (3). Dermis displays hyperemia of papillary bodies (4) and adjacent free erythrocytes (arrows). (**f**) Histological section of the constricted area marked in 2 (**d**) and 2 (**e**). The crust contains neutrophil granulocytes (1), bleeding (2) and intralesional bacteria (arrow). (**g**) Magnification of the area marked in (**f**). Coagulation necrosis of the dermal papillary bodies (1) and epidermal layers within the crust. Necrotic area is demarcated by neutrophil granulocytes (2). (**h**) Magnification of the necrotic area depicted in (**g**). Structure of the papillary bodies is still intact with visible vessel walls (arrows). Cells of the dermal (1) and epidermal layers (2) are necrotic.

These constrictions were histologically characterized by purulent-necrotizing inflammation of the epidermis with the breakdown of the epidermal basement membrane (Figure 3a). The overlying crusts contained intralesional bacteria as well as neutrophil granulocytes (Figure 3a,f). In all of these areas, the dermis was characterized by granulation tissue, extending to the central longitudinal connective tissue of the tail, as described before (Figure 3a–c). Based on the cellular composition, the occurrence and the orientation of the blood vessels and the alignment of the connective tissue fibers, the granulation tissue could be assigned to different age stages. The cell-rich granulation tissue directly underlying the epidermis was composed of mononuclear cells, sprouted non-channeled blood vessels and fibroblasts (Figure 3b). Deeper layers displayed older granulation tissue containing blood vessels, fibrocytes and collagen fibers (Figure 3c), and the deepest layers were characterized by canalized blood vessels running perpendicular to the skin surface and connective tissue fibers aligned parallel to the skin surface. Further findings in the constricted areas covered with crusts were bleedings and spongiosis (Figure 3d). In two cases, the epidermis also displayed parakeratotic hyperkeratosis and intracellular edema of the keratinocytes in the marginal area of the constrictions (Figure 3e). In the dermis, these areas were characterized by hyperemia of the papillary bodies and free erythrocytes (Figure 3e). In two cases, the crusts contained remnants of necrotic skin, with coagulation necrosis of the epidermis and the underlying papillary bodies (Figure 3f–h). Necrotic areas were demarcated by neutrophil granulocytes (Figure 3g).

### 3.2. Lesions at the Very Tip of the Tail

The macroscopic assessment revealed nine samples with lesions at the very tip of the tail. In eight cases, the tail tips were alopecic with otherwise intact skin. These alopecic areas ranged in size from 2 × 2 cm to 3 × 3 cm. The histopathological examination revealed the different age stages of the granulation tissue up to fibrosis, as described above. As with the constrictions, hair follicles and sebaceous glands were not detectable and the formation of granulation tissue extended to the central longitudinal connective tissue. The epidermis was inconspicuous in three of these areas and affected mainly by orthokeratotic and partly by parakeratotic hyperkeratosis in another four areas. In one alopecic tail tip, we found a follicle-poor area associated with two infundibular cysts.

**Figure 4:**
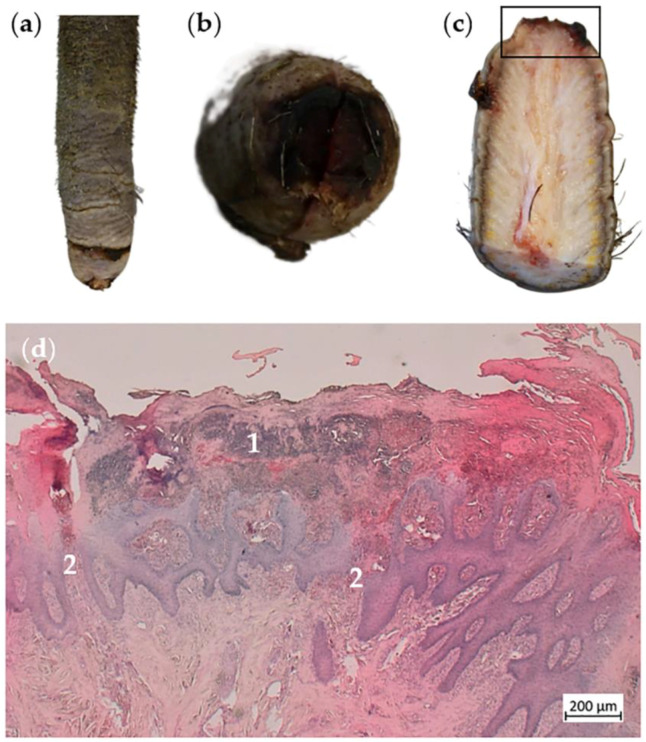
Alopecic tail with encrustation of the tail tip. (**a**) Distal part of a tail with large-scale alopecia and annular constriction encompassing about 95% of the tail circumference. (**b**) Tip of the tail depicted in (**a**) with alopecia and encrustation. (**c**) Sagittal section of the same tail with loss of tissue at the tip of the tail. (**d**) Histological section of the area marked in (**c**) with purulent-necrotizing inflammation of the epidermis and crust formation showing the accumulation of neutrophil granulocytes (1) and the multifocal breakdown of the basement membrane (2).

Another tail tip was covered by bloody crusts (Figure 4a,b). In the sagittal section, tissue loss was clearly visible at the very tip of the tail (Figure 4c). Histologically, the affected area was characterized by a shed horn layer, containing neutrophil granulocytes and intralesional bacteria (Figure 4d). The epidermis and dermis were affected by purulent-necrotizing inflammation with the breakdown of the basement membrane and ballooning degeneration of keratinocytes (Figure 4d).

Furthermore, in three of the altered tail tips, the tissue layer between the epidermis and central longitudinal connective tissue was less than 2 mm thick, while, in unaltered tail tips, the thickness of this tissue layer varied between 6 and 11 mm.

### 3.3. Scaling

**Figure 5:**
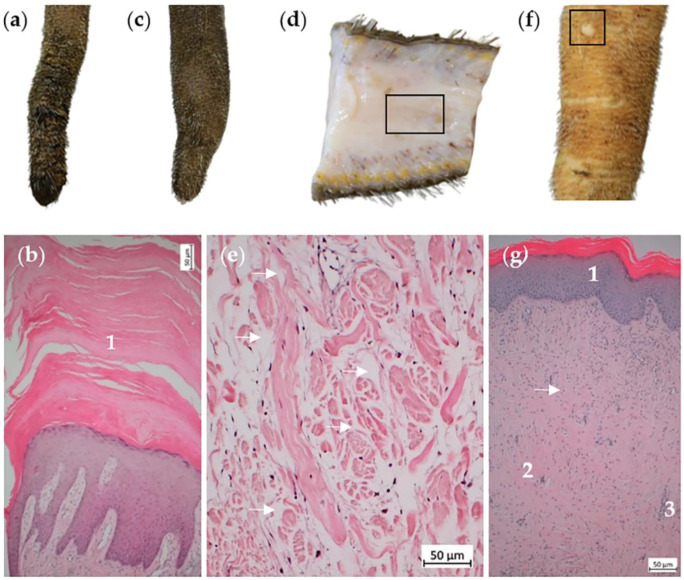
Tails with scaling, swelling or wart-like masses. (**a**) Distal part of a tail with severe scaling. (**b**) Histological section of the tail tip depicted in (**a**), H.E. Epidermis displays orthokeratotic hyperkeratosis (1). (**c**) Distal part of a tail with macroscopically visible and palpable swelling. (**d**) Sagittal section of the tail shown in (**c**). (**e**) Histological section of the area marked in (**d**), H.E. Extracellular, low-protein edema (arrows) in the region of the central longitudinal arranged connective tissue. (**f**) Distal part of a tail with single wart-like mass. (**g**) Histological section of the wart-like mass shown in (**f**), H.E. Inconspicuous epidermis (1) with underlying layer of fibroblasts (arrow), connective tissue matrix (2) and blood vessels (3).

Macroscopically, scaling was observed in four tails (Figure 5a). In one of these tails, no hyperkeratosis could be detected histologically. The three other tails were characterized predominantly by orthokeratotic (Figure 5b) and partially by parakeratotic hyperkeratosis. Furthermore, we observed orthokeratotic and partially parakeratotic hyperkeratosis in two macroscopically inconspicuous tails. In one tail with severe scaling, we additionally found bifocal purulent-necrotizing epidermitis with basement membrane breakdown and hemorrhage. The adjacent tissue revealed no granulation tissue.

### 3.4. Swelling

We found macroscopically visible swelling in eight tails (Figure 5c,d). Histologically, the edema in the area of the central longitudinal connective tissue presented as extracellular and low-protein edema since it was not stainable by H.E. (Figure 5e). We detected intracellular edema, as well as spongiosis of the epidermis only in the marginal area of the above-described bloody encrustations of constrictions and hairless areas.

### 3.5. Wart-like Masses

We found wart-like masses in seven tails (Figure 5f). These were between about 2 × 2 × 2 mm and 4 × 3 × 3 mm, being large, prominent, alopecic, well-circumscribed, nonencapsulated dermal nodes. In three of the tails, multiple wart-like masses were found. Histologically, the nodules were covered by an unaltered epidermis and consisted of abundant collagen, fibroblasts and blood vessels. No hair follicles or sebaceous glands were detected in the central areas of the masses. The collagen fibers were arranged in a dense pattern of repetitive collagen. Therefore, the masses were diagnosed as fibromas (Figure 5g). In the skin layer underneath the masses, hair follicles could be seen.

### 3.6. Narrowing of the Tail

The narrowing of the tail describes a macroscopically visible reduction in the tail diameter. This was evident in four tails. Histologically, we observed a generalized reduction in all layers of the skin, as well as the central connective tissue of the tail. Two thinnings followed a proximal constriction.

### 3.7. Additional Histopathological Findings

All tails showed the minor to moderate perivascular accumulation of predominantly mononuclear immune cells. One tail showed eosinophil granulocytes additionally. In 10 tails, the histopathological examination revealed focal (two tails), oligofocal (four tails), multifocal (three tails) or diffuse (one tail) hyperemia in the area of the papillary bodies (Figure 3e). In three of these tails, we also detected focally free erythrocytes (Figure 3e). We could not detect evidence of inflammation in the blood vessels or in the tissue of the papillary bodies, such as the cellular involvement of neutrophil granulocytes, inflammatory cells in the vessel walls, vessel wall necrosis, thrombi or fibrin adhesions. In two tails, we found generalized regular epidermal hyperplasia; in two other tails, regular epidermal hyperplasia was detected in the marginal area of a constriction.

## 4. Discussion

In a previous study on the frequency of tail tip lesions in dairy cows, we found an overall percentage of 86.7% in Holstein and 97.8% in Simmental cows [7]. This frequent occurrence of lesions raised the question of their cause. Therefore, we preselected tail tips at the abattoir for observable changes in order to elucidate the histopathological changes underlying the whole range of gross lesions. Since the selected samples originated from German Holstein, Simmental and Brown Swiss cows and an unknown breed, the issue of tail tip lesions appears to be a phenomenon that occurs across different breeds. However, given the small sample size of 16 tails examined, no conclusions can be drawn about the predisposition of different breeds to such injuries. Because the housing conditions of the animals in the sample examined were not known, it was also not possible to determine whether the injuries were caused by specific predisposing environmental factors. Therefore, the observed findings cannot be placed in the context of specific housing conditions or related to specific breeds.

The selection of the tail specimens at the abattoir was based on macroscopic findings, with the aim to ensure that all of the lesions described so far [7] were present in the sample. We were able to find all of the various lesions during the sampling at the abattoir, indicating that there is a diverse spectrum of tail alterations in dairy cows. Thus, classifications such as those used by Cuttance et al., which categorize tail lesions in dairy cows as deviated, shortened or traumatic, might not be sufficiently detailed [5].

The most frequently observed histopathological finding in our study was dermal granulation tissue formation at different stages with the loss of hair follicles and sebaceous glands. This was the case both in alopecic areas and in annular constrictions. Dermal scarring and follicular atrophy were also described in feedlot cattle affected by tail tip alopecia and necrosis [1]. Granulation tissue and fibrosis are stages of nonspecific wound healing of the skin and do not allow any conclusion about the primary noxious agent [8]. In the majority of our samples, dermal scarring was present in areas with alopecic but otherwise intact skin, without any further external sign of trauma or scar formation and no involvement of inflammatory cells such as neutrophil granulocytes. We assume that, in these cases, the lesion in the epidermis had already healed, while the dermis in this area had been permanently replaced by granulation tissue. Since granulation tissue does not contain hair follicles, this results in alopecia at the macroscopic level. This is consistent with the observations of Volhøj et al., who described signs of secondary wound healing such as granulation tissue with the loss of hair follicles and sebaceous glands in so-called “band-shaped” lesions on the tail tips of Holstein cows [6]. In our study, in areas where annular constrictions or alopecia were associated with bloody crusts, we observed purulent-necrotizing inflammation of the epidermal and dermal layers with masses of neutrophil granulocytes, in addition to the young, cell-rich granulation tissue of the dermis. This combination suggests that the primary cause of the lesion occurred some time ago, while the wound surface was still subject to irritation and thus the inflammation remained florid. In some cases, the crusts contained bacterial lawn, which might have caused the continuing inflammation.

Necrosis with underlying granulation tissue formation has also been observed in the skin of the tails of feedlot cattle suffering from intoxication with ergot alkaloids [9,10]. Intoxication with these mycotoxins leads to arterial vasoconstriction followed by endothelial damage and thrombosis [11]. The resulting ischemia causes dry gangrene in the tails, hooves and ear tips of affected cattle [9,10]. Thus, in these cases, the primary cause of the granulation tissue formation is ischemia of the skin and subsequent necrosis. The samples examined in the course of this study were collected at an abattoir and therefore originated from different farms that were not further characterized. Thus, we did not have any data on feeding management and could not draw any conclusions as to the mycotoxin loads of the feed of the selected cows or the possible implications of mycotoxin intake for the observed lesions. In pigs, the direct or indirect involvement of mycotoxins in the pathogenesis of the recently described swine inflammation and necrosis syndrome (SINS), resulting in lesions of the tails and other body parts, has been discussed [12]. Thus, future research should address this issue also in dairy cows.

Ischemia of the skin as the primary cause of tail lesions has also been suggested in other studies [1,13]. George et al. found that the local treatment of the tails of buffaloes suffering from tail inflammation and necrosis with a vasodilative substance resulted in complete recovery when applied at an early stage [13]. In the context of ringtail in laboratory rodents, necrosis of the tail tip is also caused by circulatory disturbances and consequent ischemia [14,15]. In this case, disorder in cornification results in a keratin ring, which encircles the tail and thereby causes the compression of the underlying soft tissue and finally the ischemic necrosis of the tail distal to the constriction [15]. In dairy cows, one possible explanation for ischemic necrosis might be the use of tapes or nylons, which are tied to the tails of cows to mark them. This could possibly lead to the compression of the underlying tissue. However, the use of tapes on the tails is not very common in Southern Germany, where the samples were collected. We therefore consider it unlikely that tapes are the cause of the ischemia and resulting necrosis in dairy cows. In the tails of mice affected by ringtail, histopathological findings include ortho- and parakeratotic hyperkeratosis and dilatation and thrombosis of the dermal blood vessels; in severe cases, this is accompanied by dermal hemorrhage [14]. We did not find any causes of ischemia such as thrombi or fibrin adhesions in the blood vessels in any of our samples. This might have been due to the timing of the examination. The presence of older granulation tissue in the deeper layers indicated that the lesions were not acute but that the primary noxious agent was encountered some time ago. This is in accordance with the above-mentioned study on so-called “band-shaped lesions” in Holstein cows, which describes all of the observed lesions as chronic [6]. Therefore, any thrombi present in the acute phase may have already been dissolved at the time of sampling. As our samples were taken from slaughtered dairy cows, it was not possible to investigate the progression of the lesions over time. However, this question should be investigated further in the future. We observed coagulation necrosis of the epidermal and dermal layers in the crusts of tails with annular constrictions, which points towards ischemia as the cause of the lesions. We observed these necrotic remnants in only two of the samples examined, which may have been due to the fact that the crusts are often lost during the processing of samples for histopathological examination. Additionally, we found that in tails with macroscopic narrowing, all layers of the tail were equally narrowed. This could also be due to poor blood circulation. However, histopathological examination alone does not allow us to draw a final conclusion as to the cause of the observed narrowing.

Disturbances in blood circulation are also responsible for causing laminitis in cows [16,17]. Several studies suggest a link between ruminal acidosis and laminitis as endotoxins and vasoactive substances released in the course of ruminal acidosis are thought to cause damage to the digital microvasculature, resulting in dermal ischemia (reviewed in [18]). Since both the claws and the tail represent a vascular border zone, the similar pathogenesis of the observed tail lesions would be conceivable in principle. Interestingly, a cohort study with German Holstein and Simmental fattening bulls revealed that bulls with tail necrosis had a lower ruminal pH than bulls with inconspicuous tail tips [19], supporting this hypothesis. The ortho- and parakeratotic hyperkeratosis that we observed in several tails might therefore be the result of disturbed blood flow in the dermal layers. Hyperkeratosis has also been described in the tail tips of fattening cattle with alopecia and crustiness [1]. While we ruled out dermatophytes and ectoparasites as the cause of hyperkeratosis via the periodic acid–Schiff reaction, this hypothesis needs to be tested in future studies. Histopathological findings in the claws of cattle affected by laminitis include hyperemia, edema, thrombosis and hemorrhages in the dermis; the detachment of the basement membrane; and, in chronic cases, granulation tissue and fibrosis, as well as ortho- and parakeratotic hyperkeratosis [16,20,21]. Thus, they closely resemble the histopathological findings in our samples, with the exception of thrombosis. In our study, low-protein edema was present in the central longitudinal connective tissue in eight samples. This might have been due to disturbances in blood flow, as is the case in laminitis; however, the exact underlying pathophysiological mechanisms merit further investigation. In the tails of fattening cattle, edema in and around the vessel walls has been described [1], but the diffuse edematous swelling of the central longitudinal connective tissue is—to our knowledge—a new observation. A case report study on a dairy farm in Israel reported on a lameness–dermatitis syndrome as a result of the sudden addition of a large quantity of readily fermentable carbohydrate to the feed rations of pregnant heifers [22]. Lesions of the skin in the digits and tails appeared simultaneously, and the histopathological changes in the tails included necrotic dermatitis, hyperkeratosis and dermal edema [22]. Since both dairy cows and fattening cattle are often fed high-concentrate ratios with a high degree of readily fermentable carbohydrate, future studies should investigate the relationship between ruminal acidosis, laminitis and tail lesions in both dairy and fattening cattle.

In pigs, inflammation and necrosis of the tails, ears, claws and other body parts have been reported as a symptom of SINS [23,24]. SINS is considered a general inflammatory response with changes in local blood circulation, which might then lead to ischemic necrosis, especially in the acra [25]. Because symptoms can be noted immediately after birth, the tail tip inflammation in this context is thought to have an endogenous cause and is not due to external factors such as biting or mechanical irritation [26]. In this study, lesions were observed in tails from cows of presumably different ages. Since the samples were collected at an abattoir, it was not possible to examine the tails of newborn calves for comparison. Thus, we cannot rule out that the observed lesions were the result of external trauma with subsequent infection, as some authors have argued in the case of feedlot cattle [3,4]. In dairy cows, several scenarios that would result in trauma to the tail are possible, like trampling or injuries caused by the scraper or other barn facilities and also the use of tapes. Noticeably, in all observed stages of the dermal granulation tissue in our study, the changes extended to the central longitudinal tissue of the tail, rendering it unlikely that these changes were a consequence of external factors causing injury to the skin. However, due to the origin of our samples, we had no information regarding the housing system or the history of the lesions and therefore we cannot rule out that the lesions were caused by external factors. Volhoj et al. also concluded that the band-shaped lesions examined could be due to disturbed blood flow in the tail tip, as the segmented appearance of the lesions described could reflect the architecture of the blood vessels in the tail [6]. However, based on our findings, we cannot draw any conclusions about the primary cause of the cutaneous ischemia of the tail tips. The histopathological findings of piglets with SINS are characterized by subepithelial infiltration with granulocytes and lymphocytes and perivascular edema together with an intact epidermis in most cases [26]. All of the tails examined in our study showed the perivascular accumulation of mononuclear cells such as lymphocytes and macrophages. However, the perivascular accumulation of inflammatory cells has been described as a physiological state in bovine tails [27,28], since this finding occurs in the macroscopically inconspicuous tails of adult and newborn animals. Therefore, the clinical relevance of this finding is unclear to date. It was suggested that the onset of SINS might be due to microbe-associated molecular patterns such as lipopolysaccharide, which enter the bloodstream mainly via a leaky gut and thereby cause a systemic inflammatory reaction [12]. It is known that dairy cows undergo a state of systemic inflammation during the transition period [29,30]. Therefore, a similar pathomechanism would be conceivable and should be investigated in future studies. If it is the case that excessive or chronic systemic inflammation leads to ischemia in the blood vessels of the acra, including the tail tip, and subsequent necrosis, the examination of the easily accessible tip of the tail might be a valuable tool in the future for the assessment of the health status of dairy cows. Thus, future studies should investigate whether there is an association between the inflammatory state and the observed tail tip lesions.

A further result of this study is that the observed wart-like masses on the tails were fibromas. Collagenous hamartoma and skin tags can be considered as differential diagnoses. However, hair follicles and glands are usually found in skin tags and collagenous hamartomas are usually poorly circumscribed and the collagen fibers are arranged haphazardly. Fibromas are benign neoplasms that are uncommon in cattle and rarely described in the literature [31]. Future studies should therefore investigate whether there is a relationship between fibromas and the other lesions observed or whether fibromas of the tail represent a separate disorder.

## 5. Conclusions

The histopathological appearance of the tail tips examined in this study resembled that of tail tip necrosis in fattening cattle. The histopathological findings such as granulation tissue formation, hyperkeratosis, hyperemia, edema and hemorrhages are consistent with previous suggestions of cutaneous ischemia, but they do not allow definitive conclusions regarding the underlying cause of the lesions. However, our observation of coagulation necrosis in the epidermal and dermal layers of encrusted tail tips supports the hypothesis of ischemia of the skin as the cause of the necrosis of the tail tips. Further studies are required to test this hypothesis and to elucidate the underlying pathomechanisms.

## Figures and Tables

**Table 1:** Overview of the macroscopic and respective histopathological findings.

Macroscopic Findings			Histopathological Findings	
Lesion	Number of Tails with Respective Lesion	Description	Number of Areas with Respective Findings	Description
Annular constrictions	10		20	Different stages of dermal scarring
		Alopecic skin	8	No epidermal abnormality detected
			4	Orthokeratotic hyperkeratosis
			2	Parakeratotic hyperkeratosis
		Marginal alopecic skin, central bloody crusts	1	Marginal no epidermal abnormality detected, central florid inflammation
			2	Marginal orthokeratotic hyperkeratosis, central florid inflammation
			1	Marginal orthokeratotic hyperkeratosis, central purulent-necrotizing inflammation, crust with remnants of necrotic skin
		Bloody crusts	1	Purulent-necrotizing inflammation
			1	Florid inflammation, crust with remnants of necrotic skin
Lesions at the very tip of the tail	9	Alopecia	3	Different stages of dermal scarring, no hair follicles, inconspicuous epidermis
			4	Different stages of dermal scarring, no hair follicles, mainly orthokeratotic, with one case of parakeratotic hyperkeratosis
			1	Lower hair follicle count, infundibular cyst, inconspicuous epidermis
		Bloody crusts	1	Purulent-necrotizing inflammation with underlying granulation tissue, no hair follicles
Scaling	2	No scaling	1	Orthokeratotic hyperkeratosis
			1	Predominantly orthokeratotic, partly parakeratotic hyperkeratosis
	4	Mild to severe scaling	1	Inconspicuous epidermis
			3	Predominantly orthokeratotic, partly parakeratotic hyperkeratosis
Swelling	8	Swelling in the region of the central longitudinal connective tissue	8	Extracellular, low-protein edema
Wart-like masses	7	Well-circumscribed, alopecic, nonencapsulated dermal nodes	12	Nodes consisting of abundant collagen, fibroblasts and blood vessels, covered by an unaltered epidermis
Narrowing of the tail	4	Narrowing of all layers of the tail	4	Narrowing of all layers of the tail

## Data Availability

Data are contained within the article.
